# Supplementary material for: Limits on the evolutionary rates of biological traits
Source: Sci Rep. 2024 May 17;14:11314. doi: 10.1038/s41598-024-61872-z (PMC11101453; doi:10.1038/s41598-024-61872-z)
Supplement: Supplementary file 1 — Supplementary Information. [file 41598_2024_61872_MOESM1_ESM.pdf]

Supplementary Material
Limits on the Evolutionary Rates of Biological Traits

Luis Pedro García-Pintos[1,2,*]

[1] Theoretical Division (T4), Los Alamos National Laboratory, Los Alamos, New Mexico 87545, USA
[2] Joint Center for Quantum Information and Computer Science and Joint Quantum Institute, NIST/University of Maryland, College Park, Maryland 20742, USA

This Supplementary Material includes detailed proofs of Eqs. (1), (2), (9), (11), (14), and (15) in the main text. I also show that the techniques developed in this article can be applied to other dynamical models that incorporate stochastic forces, and illustrate the rate limits for the replicator-mutator equation on a toy model.

I. RATE LIMITS FOR ARBITRARY EVOLUTIONARY DYNAMICS

I prove Eqs. (1) and (2) in the main text. I also show that the variance in the growth rate of a population equals the classical Fisher information of the frequency distribution of the population.

Let n_j be the time-dependent population of type j and $N := \sum_j n_j$, so that $\{p_j := n_j/N\}$ is a normalized distribution. The rate of change of the average $\langle A\rangle := \sum_j \frac{n_j}{N} a_j$ over the population satisfies

$$\frac{d}{dt}\langle A\rangle - \langle \dot{A}\rangle = \sum_j a_j \frac{\dot{n}_j}{N} - \frac{\dot{N}}{N}\sum_j a_j \frac{n_j}{N} = \sum_j a_j \frac{n_j}{N}\frac{\dot{n}_j}{n_j} - \frac{\dot{N}}{N}\langle A\rangle = \sum_j \delta a_j \frac{n_j}{N}\frac{\dot{n}_j}{n_j} + \langle A\rangle \sum_j \frac{n_j}{N}\frac{\dot{n}_j}{n_j} - \frac{\dot{N}}{N}\langle A\rangle$$
$$= \sum_j \delta a_j \frac{n_j}{N}\frac{\dot{n}_j}{n_j} = \sum_j \delta a_j \frac{n_j}{N} r_j = \sum_j \delta a_j \frac{n_j}{N}\delta r_j, \tag{1}$$

where I denote $\delta a_j := a_j - \langle A\rangle$ and $\delta r_j := r_j - \langle r\rangle$, with $r_j := \frac{\dot{n}_j}{n_j}$. In the last step I used that $\langle r\rangle \sum_j \delta a_j n_j/N = 0$ given that $\langle \delta A\rangle = 0$.

That is,

$$\frac{d}{dt}\langle A\rangle - \langle \dot{A}\rangle = \sum_j \delta a_j\, \delta r_j \frac{n_j}{N} = \mathrm{cov}(r, A), \tag{2}$$

where the covariance is defined by $\mathrm{cov}(r, A) := \langle \delta A\, \delta r\rangle$. This proves Eq. (1) in the main text.

Using that the covariance of two quantities is bounded by the product of their standard deviations, I obtain that

$$\left|\frac{d}{dt}\langle A\rangle - \langle \dot{A}\rangle\right| = |\mathrm{cov}(r, A)| \leq \sigma_r\, \sigma_A, \tag{3}$$

where $\sigma_A := \sqrt{\langle A^2\rangle - \langle A\rangle^2}$ denotes the standard deviation of A. Equation (3) implies that, for any trait A, the evolution of the mean is fast only if A or r have variability – i.e., their standard deviations σ_r and σ_A cannot be small for fast evolution. This proves Eq. (2) in the main text.

Defining the normalized frequency distribution $p_j := n_j/N$ and using that $\dot{p}_j = \dot{n}_j/N - n_j\dot{N}/N^2$, it holds that

$$\frac{\dot{p}_j}{p_j} = \frac{\dot{n}_j}{n_j} - \frac{\dot{N}}{N}. \tag{4}$$

Then,

$$\mathcal{I}_F := \sum_j p_j \left(\frac{\dot{p}_j}{p_j}\right)^2 = \sum_j \frac{n_j}{N}\left(\frac{\dot{n}_j}{n_j} - \frac{\dot{N}}{N}\right)^2 = \sum_j \frac{n_j}{N}\left(\frac{\dot{n}_j}{n_j}\right)^2 - 2\sum_j \frac{n_j}{N}\frac{\dot{n}_j}{n_j}\frac{\dot{N}}{N} + \sum_j \frac{n_j}{N}\frac{\dot{N}^2}{N^2} = \sum_j \frac{n_j}{N}\left(\frac{\dot{n}_j}{n_j}\right)^2 - \left(\frac{\dot{N}}{N}\right)^2$$
$$= \sum_j \frac{n_j}{N}\left(\frac{\dot{n}_j}{n_j}\right)^2 - \left(\sum_j \frac{n_j}{N}\frac{\dot{n}_j}{n_j}\right)^2 = \sum_j \frac{n_j}{N} r_j^2 - \left(\sum_j \frac{n_j}{N} r_j\right)^2 = \left\langle r^2\right\rangle - \langle r\rangle^2 \equiv (\sigma_r)^2\,. \tag{5}$$

* lpgp@lanl.gov

That is, the variance in the rate r equals the Fisher information of the normalized distribution $p_j := n_j/N$. Note, too, that Eq. (4) implies that $\mathrm{cov}(A, r) = \mathrm{cov}(A, f)$ for dynamics governed by the replicator equation.

II. LIMITS TO EVOLUTIONARY PROCESSES WITH MUTATIONS

In this section I prove Equations (9) and (11) in the main text.

Let $\delta_\Pi a_j := a_j - \langle A\rangle_\Pi$ and $\delta f_j := f_j - \langle f\rangle$, where $\langle A\rangle_\Pi := \sum_j \Pi_j a_j$ and $\langle f\rangle := \sum_j p_j f_j$ are means with respect to the distribution Π with components $\Pi := \sum_k p_k Q_{kj}$ and the distribution p, respectively. Then, using conservation of probability (which implies $\sum_j \dot{p}_j \langle A\rangle_\Pi = 0$) and the mutator-replicator equation, it holds that

$$\begin{aligned}
\frac{d\langle A\rangle}{dt} - \langle \dot{A}\rangle &= \sum_j \dot{p}_j a_j = \sum_j \dot{p}_j \delta_\Pi a_j \\
&= \sum_{jk} Q_{kj} f_k p_k \delta_\Pi a_j - \sum_j p_j \delta_\Pi a_j \langle f\rangle \\
&= \sum_{jk} Q_{kj} \delta f_k\, p_k\, \delta_\Pi a_j + \sum_{jk} Q_{kj} \langle f\rangle\, p_k\, \delta_\Pi a_j - \langle f\rangle\langle A\rangle + \langle f\rangle\langle A\rangle_\Pi \\
&= \sum_{jk} Q_{kj} \delta f_k\, p_k\, \delta_\Pi a_j + \langle f\rangle \sum_j \Pi_j\, \delta_\Pi a_j - \langle f\rangle\langle A\rangle + \langle f\rangle\langle A\rangle_\Pi \\
&= \sum_{jk} Q_{kj} \delta f_k\, p_k\, \delta_\Pi a_j - \langle f\rangle\big(\langle A\rangle - \langle A\rangle_\Pi\big).
\end{aligned} \tag{6}$$

The Cauchy-Schwarz inequality says that $\sum_\alpha X_\alpha Y_\alpha \le \sqrt{(\sum_\alpha X_\alpha^2)(\sum_\alpha Y_\alpha^2)}$. Applying it to the first term in the last line, with $X_\alpha = \sqrt{Q_{kj}}\delta f_k \sqrt{p_k}$ and $Y_\alpha = \sqrt{Q_{kj} p_k}\delta_\Pi a_j$ where α denotes both indexes $\{j,k\}$, gives

$$\begin{aligned}
\left(\sum_{jk} Q_{kj}\delta f_k\, p_k\, \delta_\Pi a_j\right)^2 &\le \left(\sum_{jk} Q_{kj}\,(\delta f_k)^2\, p_k\right)\left(\sum_{jk} Q_{kj} p_k\,(\delta_\Pi a_j)^2\right) \\
&= \left(\sum_k (\delta f_k)^2\, p_k\right)\left(\sum_j \Pi_k\,(\delta_\Pi a_j)^2\right) = (\sigma_f)^2\,\left(\sigma_A^\Pi\right)^2,
\end{aligned} \tag{7}$$

where I used that $\sum_j Q_{kj} = 1$. Combining Eqs. (6) and (7) leads to

$$\left|\frac{d\langle A\rangle}{dt} - \langle\dot{A}\rangle - \langle f\rangle\Big(\langle A\rangle_\Pi - \langle A\rangle\Big)\right| = \left|\sum_{jk} Q_{kj}\delta f_k\, p_k\, \delta_\Pi a_j\right| \le \sigma_A^\Pi\,\sigma_f, \tag{8}$$

which proves Equation (9) in the main text.

Choosing a trait with components $a_j \equiv I_j = -\ln p_j$ gives:

$$\langle A\rangle = \sum_j p_j(-\ln p_j) = S \tag{9}$$

$$\langle A\rangle_\Pi - \langle A\rangle = \sum_j \Pi_j(-\ln p_j) - \sum_j p_j(-\ln p_j) = S(p||\Pi). \tag{10}$$

Using that $\langle\dot{A}\rangle = \sum_j p_j(-\dot{p}_j/p_j) = -\sum_j \dot{p}_j = 0$ yields Equation (11) in the main text.

III. LIMITS TO STOCHASTIC EVOLUTIONARY PROCESSES

In this section I prove Equation (14) in the main text.

Consider a stochastic replicator-mutator equation,

$$dp_j = \sum_k p_k Q_{kj}\big(f_k - \langle f\rangle\big)dt + p_j\left(\gamma_j dW_j - \sum_l \gamma_l p_l dW_l\right), \tag{11}$$

where γ_j characterizes the strength of the stochastic driving forces for population j, and dW_j are Wiener noises, which satisfy $\overline{dW_j dW_k} = \delta_{jk} dt$ and $dW_j^2 = dt$.

Let us focus on the noise-averaged change in an expectation value over a time τ relative to the change

$$\big[d\langle A\rangle - \langle dA\rangle\big]\Big|_{\text{replicator-mutator}} = \sum_j a_j dp_j\Big|_{\text{replicator-mutator}} = \sum_{jk} p_k Q_{kj}\big(f_k - \langle f\rangle\big) a_j dt \tag{12}$$

in $\langle A\rangle$ due to state changes from natural selection and mutations, as modeled by the replicator-mutator equation. It is given by

$$\begin{aligned}
\overline{\left|\frac{1}{\tau}\int_0^\tau \left(d\langle A\rangle - \langle dA\rangle - \sum_{jk} p_k Q_{kj}\big(f_k - \langle f\rangle\big) a_j dt\right)\right|^2} &= \overline{\left|\frac{1}{\tau}\int_0^\tau \left(\sum_j dp_j a_j - \sum_{jk} p_k Q_{kj}\big(f_k - \langle f\rangle\big)\delta a_j dt\right)\right|^2} \\
&= \overline{\left|\frac{1}{\tau}\int_0^\tau \left(\sum_j \delta a_j \big(dp_j - \sum_k p_k Q_{kj}\big(f_k - \langle f\rangle\big)dt\big)\right)\right|^2} \\
&= \overline{\left|\frac{1}{\tau}\int_0^\tau \sum_j \delta a_j p_j \left(\gamma_j dW_j - \sum_l \gamma_l p_l dW_l\right)\right|^2} \\
&= \overline{\left|\frac{1}{\tau}\int_0^\tau \sum_j \delta a_j p_j Y_j\right|^2} \\
&= \frac{1}{\tau^2}\int_0^\tau\int_0^\tau \sum_{jk} \overline{\delta a_j \delta a_k p_j p_k Y_j Y_k},
\end{aligned} \tag{13}$$

where I used that $\delta a_j \coloneqq a_j - \langle a\rangle$, that probability is conserved, $\sum_j dp_j = 0$, and Eq. (11). Here,

$$Y_j \coloneqq \left(\gamma_j dW_j - \sum_l \gamma_l p_l dW_l\right). \tag{14}$$

In order to prevent confusion, I explicitly include the time-dependence of the noise terms. For Wiener processes, it holds that $\overline{dW_j^t dW_j^{t'}} = \delta_{jk}\delta_{tt'}$, and that the noise terms are independent from all other functions (in the Itô picture), so that $\overline{p_j^t dW_j^t} = \overline{p_j^t}\ \overline{dW_j^t} = 0$. The integrals that lack a line element dt, proportional to a noise term, correspond to Itô integrals [1].

Using the rules of Itô calculus described above and that $\sum_j \delta a_j p_j = 0$ gives that

$$\begin{aligned}
\int_0^\tau\int_0^\tau \sum_{jk} \overline{\delta a_j \delta a_k p_j p_k Y_j Y_k} &= \int_0^\tau\int_0^\tau \sum_{jk} \overline{\delta a_j \delta a_k p_j p_k \gamma_j \gamma_k dW_j^t dW_k^{t'}} \\
&- 2\int_0^\tau\int_0^\tau \sum_{jk} \overline{\delta a_j \delta a_k p_j p_k \gamma_j dW_j^t \sum_l p_l \gamma_l dW_l^{t'}} \\
&+ \int_0^\tau\int_0^\tau \sum_{jk} \overline{\delta a_j \delta a_k p_j p_k \left(\sum_l p_l \gamma_l dW_l^t\right)\left(\sum_m p_m \gamma_m dW_m^{t'}\right)} \\
&= \int_0^\tau \sum_j \overline{(\delta a_j)^2 \gamma_j^2 p_j^2} dt - 2\int_0^\tau \sum_{jk} \overline{\delta a_j \delta a_k p_j p_k \gamma_j p_j \gamma_j} dt \\
&+ \int_0^\tau \sum_{jk} \overline{\delta a_j \delta a_k p_j p_k \left(\sum_l p_l^2 \gamma_l^2\right)} dt \\
&= \overline{\int_0^\tau \sum_j (\delta a_j)^2 \gamma_j^2 p_j^2 dt}.
\end{aligned} \tag{15}$$

Combining Eq. (13) and (15) results in

$$\overline{\left(\frac{1}{\tau}\int_0^\tau d\langle A\rangle - \langle dA\rangle - \sum_{jk} p_k Q_{kj}\big(f_k - \langle f\rangle\big)a_j dt\right)^2} = \frac{1}{\tau^2}\overline{\int_0^\tau \sum_j (\delta a_j)^2\,\gamma_j^2 p_j^2 dt}$$
$$\leq \|\gamma\|_\infty^2 \frac{1}{\tau^2}\overline{\int_0^\tau \sum_j (\delta a_j)^2\, p_j^2 dt}$$
$$\leq \|\gamma\|_\infty^2 \frac{1}{\tau^2}\overline{\int_0^\tau \sum_j (\delta a_j)^2\, p_j dt}$$
$$= \frac{\|\gamma\|_\infty^2}{\tau}\frac{1}{\tau}\overline{\int_0^\tau (\sigma_A)^2 dt}. \tag{16}$$

This follows from $\gamma_j \leq \|\gamma\|_\infty := \max_j\{\gamma_j\}$ and $p_j^2 \leq p_j$. This proves Eq. (14) in the main text.

A. Limits under other stochastic dynamics

An alternative stochastic replicator equation of the form

$$dp_j = p_j\left(f_j - \langle f\rangle - \gamma_j^2 p_j + \sum_l \gamma_l^2 p_l^2\right)dt + p_j\left(\gamma_j dW_j - \sum_l \gamma_l p_l dW_l\right) \tag{17}$$

was derived in Ref. [2]. For simplicity, I assume no mutations in this sub-appendix, but it is easy to generalize the results that follow to account for them. Here, γ_j characterizes the strength of the stochastic driving forces for population j, and dW_j are Wiener noises, which satisfy $\overline{dW_j dW_k} = \delta_{jk}dt$ and $dW_j^2 = dt$.

Consider the noise-averaged change in an expectation value over a time τ, relative to the change $\big[d\langle A\rangle - \langle dA\rangle\big]\Big|_{\text{replicator}} = \text{cov}(A, f$ in $\langle A\rangle$ due state changes from natural selection as modeled by the replicator equation.

Following similar calculations as above, one finds that all cross terms proportional to a single noise term vanish upon averaging, and therefore

$$\overline{\left(\frac{1}{\tau}\int_0^\tau d\langle A\rangle - \langle dA\rangle - \text{cov}(A,f)dt\right)^2} = \frac{1}{\tau^2}\int_0^\tau\int_0^\tau \sum_{jk}\overline{\delta a_j \delta a_k\,(dp_j - p_j\delta f_j dt)\,(dp_k - p_k \delta f_k dt')}$$
$$= \frac{1}{\tau^2}\int_0^\tau\int_0^\tau \sum_{jk}\overline{\delta a_j \delta a_k\,(p_j X_j - p_j\delta f_j)\,(p_k X_k - p_k \delta f_k)}dtdt'$$
$$+\frac{1}{\tau^2}\int_0^\tau\int_0^\tau \sum_{jk}\overline{\delta a_j \delta a_k p_j p_k Y_j Y_k}, \tag{18}$$

by using that $dp_j = p_j X_j dt + p_j Y_j$, with the notation

$$X_j := \left(f_j - \langle f\rangle - \gamma_j^2 p_j + \sum_l \gamma_l^2 p_l^2\right) = \left(\delta f_j - \gamma_j^2 p_j + \sum_l \gamma_l^2 p_l^2\right) \tag{19}$$

$$Y_j := \left(\gamma_j dW_j - \sum_l \gamma_l p_l dW_l\right). \tag{20}$$

Then, using the rules of Itô calculus described above and that $\sum_j \delta a_j p_j = 0$ gives that the second term is

$$\begin{aligned}\int_0^\tau \int_0^\tau \sum_{jk} \overline{\delta a_j \delta a_k p_j p_k Y_j Y_k} &= \int_0^\tau \int_0^\tau \sum_{jk} \overline{\delta a_j \delta a_k p_j p_k \gamma_j \gamma_k dW_j^t dW_k^{t'}} \\ &- 2\int_0^\tau \int_0^\tau \sum_{jk} \overline{\delta a_j \delta a_k p_j p_k \gamma_j dW_j^t \sum_l p_l \gamma_l dW_l^{t'}} \\ &+ \int_0^\tau \int_0^\tau \overline{\sum_{jk} \delta a_j \delta a_k p_j p_k \left(\sum_l p_l \gamma_l dW_l^t\right)\left(\sum_m p_m \gamma_m dW_m^{t'}\right)} \\ &= \int_0^\tau \sum_j \overline{\left(\delta a_j\right)^2 \gamma_j^2 p_j^2 dt} - 2\int_0^\tau \sum_{jk} \overline{\delta a_j \delta a_k p_j p_k \gamma_j p_j \gamma_j dt} \\ &+ \int_0^\tau \overline{\sum_{jk} \delta a_j \delta a_k p_j p_k \left(\sum_l p_l^2 \gamma_l^2\right)} dt \\ &= \overline{\int_0^\tau \sum_j \left(\delta a_j\right)^2 \gamma_j^2 p_j^2 dt}. \end{aligned} \tag{21}$$

Meanwhile, the first term becomes

$$\begin{aligned}&\frac{1}{\tau^2}\int_0^\tau \int_0^\tau \sum_{jk} \overline{\delta a_j \delta a_k \left(p_j X_j - p_j \delta f_j\right)\left(p_k X_k - p_k \delta f_k\right)} dt dt' \\ &= \int_0^\tau \int_0^\tau \sum_{jk} \delta a_j \delta a_k p_j p_k \left(-\gamma_j^2 p_j + \sum_l \gamma_l^2 p_l^2\right)\left(-\gamma_k^2 p_k + \sum_m \gamma_m^2 p_m^2\right) dt dt' \\ &= \int_0^\tau \int_0^\tau \left(\left(\sum_j p_j^2 \gamma_j^2 \delta a_j\right)\left(\sum_k p_k^2 \gamma_k^2 \delta a_k\right)\right) dt dt' \\ &= \left[\int_0^\tau \left(\sum_j p_j^2 \gamma_j^2 \delta a_j\right) dt\right]^2, \end{aligned} \tag{22}$$

where I used that $\sum_j p_j \delta a_j = 0$ in the third line.

Combining the previous two equations gives

$$\begin{aligned}\overline{\left(\frac{1}{\tau}\int_0^\tau d\langle A\rangle - \langle dA\rangle - \mathrm{cov}(A,f)dt\right)^2} &= \frac{1}{\tau^2}\overline{\left(\int_0^\tau \sum_j p_j^2 \gamma_j^2 \delta a_j dt\right)^2} + \frac{1}{\tau^2}\overline{\int_0^\tau \sum_j \left(\delta a_j\right)^2 \gamma_j^2 p_j^2 dt} \\ &\le \frac{1}{\tau^2}\overline{\left(\int_0^\tau \sqrt{\sum_j p_j^2 \gamma_j^4}\sqrt{\sum_k p_k^2 \left(\delta a_k\right)^2} dt\right)^2} + \|\gamma\|_\infty^2 \frac{1}{\tau^2}\overline{\int_0^\tau \sum_j \left(\delta a_j\right)^2 p_j^2 dt} \\ &\le \frac{1}{\tau^2}\overline{\left(\int_0^\tau \sqrt{\sum_j p_j \gamma_j^4}\sqrt{\sum_k p_k \left(\delta a_k\right)^2} dt\right)^2} + \|\gamma\|_\infty^2 \frac{1}{\tau^2}\overline{\int_0^\tau \sum_j \left(\delta a_j\right)^2 p_j dt} \\ &= \overline{\left(\frac{1}{\tau}\int_0^\tau \sigma_A \sqrt{\langle\gamma^4\rangle} dt\right)^2} + \frac{\|\gamma\|_\infty^2}{\tau}\overline{\frac{1}{\tau}\int_0^\tau \left(\sigma_A\right)^2 dt}. \end{aligned} \tag{23}$$

The second line follows from the Cauchy-Schwarz inequality and from $\gamma_j \le \|\gamma\|_\infty := \max_j\{\gamma_j\}$, and the third line holds because $p_j^2 \le p_j$.

Note that the first term dominates the upper bound for long times $\tau \gg \|\gamma\|_\infty^2$.

A looser bound holds by using that $\langle\gamma^4\rangle \le \|\gamma\|_\infty^4$,

$$\overline{\left(\frac{1}{\tau}\int_0^\tau d\langle A\rangle - \langle dA\rangle - \mathrm{cov}(A,f)dt\right)^2} \le \|\gamma\|_\infty^4 \overline{\left(\frac{1}{\tau}\int_0^\tau \sigma_A\, dt\right)^2} + \frac{\|\gamma\|_\infty^2}{\tau}\overline{\frac{1}{\tau}\int_0^\tau \left(\sigma_A\right)^2 dt}. \tag{24}$$

This proves an equation analogous to Eq. (14) in the main text for the stochastic replicator equation (17).

An analogous constraint holds when mutations are included,

$$dp_j = p_j \left(\sum_k p_k f_k Q_{kj} - p_j \langle f \rangle - \gamma_j^2 p_j + \sum_l \gamma_l^2 p_l^2 \right) dt + p_j \left(\gamma_j dW_j - \sum_l \gamma_l p_l dW_l \right), \tag{25}$$

in which case

$$\overline{\left(\frac{1}{\tau} \int_0^\tau d\langle A \rangle - \langle dA \rangle - \sum_{jk} p_k (f_k - \langle f \rangle) Q_{kj} a_j dt \right)^2} \leq \|\gamma\|_\infty^4 \overline{\left(\frac{1}{\tau} \int_0^\tau \sigma_A \, dt \right)^2} + \frac{\|\gamma\|_\infty^2}{\tau} \overline{\frac{1}{\tau} \int_0^\tau (\sigma_A)^2 dt}. \tag{26}$$

IV. RATE LIMITS UNDER NATURAL SELECTION, MUTATIONS, AND GENETIC DRIFT

I prove Equation (15) in the main text.

It holds that

$$\begin{aligned}
\overline{\left| \int_0^\tau \Big(d\langle A \rangle - \langle dA \rangle \Big) \right|} - \overline{\left| \int_0^\tau \sum_{jk} p_k Q_{kj} \big(f_k - \langle f \rangle \big) a_j dt \right|} &\leq \overline{\left| \int_0^\tau d\langle A \rangle - \langle dA \rangle - \sum_{jk} p_k Q_{kj} \big(f_k - \langle f \rangle \big) a_j dt \right|} \\
&\leq \sqrt{\overline{\left| \int_0^\tau d\langle A \rangle - \langle dA \rangle - \sum_{jk} p_k Q_{kj} \big(f_k - \langle f \rangle \big) a_j dt \right|^2}} \\
&\leq \|\gamma\|_\infty \sqrt{\int_0^\tau \overline{(\sigma_A)^2} dt}
\end{aligned} \tag{27}$$

where I used the triangle inequality in the first line, the Cauchy-Schwarz inequaltiy in the second line, and bound (16) in the third line.

The rate limit (8), derived for the replicator-mutator equation, says that the replicator-mutator contribution to the change in A satisfies

$$\left| \sum_{jk} p_k Q_{kj} \big(f_k - \langle f \rangle \big) a_j \right| = \left| \left[\frac{d\langle A \rangle}{dt} - \langle \dot{A} \rangle \right] \Big|_{\text{replicator-mutator}} \right| \tag{28}$$

$$\leq \langle f \rangle \big| \langle A \rangle_\Pi - \langle A \rangle \big| + \sigma_A^\Pi \sigma_f. \tag{29}$$

Then,

$$\overline{\left| \int_0^\tau \Big(d\langle A \rangle - \langle dA \rangle \Big) \right|} \leq \overline{\int_0^\tau \sigma_A^\Pi \sigma_f dt} + \overline{\int_0^\tau \langle f \rangle \big| \langle A \rangle_\Pi - \langle A \rangle \big| dt} + \|\gamma\|_\infty \sqrt{\overline{\int_0^\tau (\sigma_A)^2 \, dt}}. \tag{30}$$

This proves Eq. (15) in the main text.

V. MATHEMATICAL TOY MODELS TO ILLUSTRATE THE BOUNDS

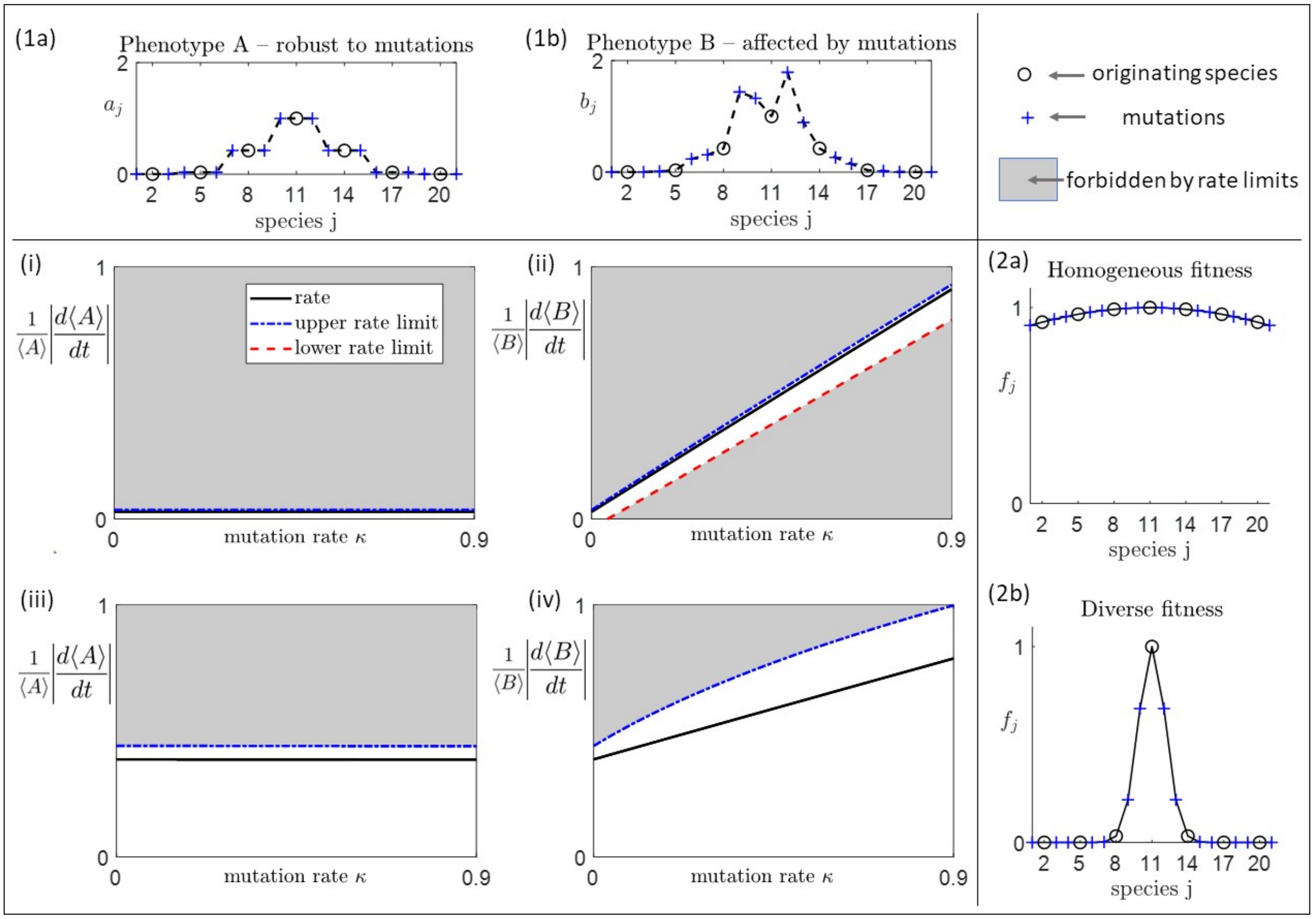

FIG. 1. **Evolutionary rate limits.** I consider a toy model where $N = 7$ types, initially with equal populations, mutate to two neighboring variants $j \longrightarrow \{j-1, j+1\}$ at rates $Q_{j,j-1} = 0.1\kappa$ and $Q_{j,j+1} = \kappa$. While the replicator-mutator Eq. (6) governs the evolution of the populations, the lower and upper rate limits Eq. (9a) and (9b) constrain the dynamics of the types' quantitative traits in terms of expectation values and standard deviations. For illustration purposes, I consider a phenotype A (e.g., the flagella length of bacteria) whose values are not affected by the mutations [inset (1a)] and a phenotype B (e.g., bacteria's mass) that changes on the mutated types [inset (1b)]. [Left column] For the mutation-robust phenotype A, it holds that $\langle A\rangle = \langle A\rangle_\Pi$, so the upper rate limit Eq. (9b) implies that $|d\langle A\rangle/dt| \leq \sigma_A^\Pi \sigma_f$. Then, a homogeneous fitness landscape (weak-selection regime) for which $\sigma_f \ll 1$ [inset (2a)] results in slower changes in the phenotype [plot (i)] than the fast rates [plot (iii)] obtained with a diverse fitness profile (natural selection regime) for which $\sigma_f \gg 1$ [inset (2b)]. [Center column] The mutation-sensitive phenotype B can evolve more rapidly than phenotype A due to the contribution of the term $\langle f\rangle|\langle B\rangle_\Pi - \langle B\rangle|$ to the upper rate limit Eq. (9b). When the mutation-driven term $\langle f\rangle\,|\langle B\rangle_\Pi - \langle B\rangle|$ is larger than the natural selection contribution $\sigma_A^\Pi\sigma_f$, the lower rate limit Eq. (9a) further constrains the minimum rates [plot (ii)]. The lower rate limits do not appear plots (i,iii,iv) since they are negative in those regimes. This example illustrates how the rate limits derived in this paper can be used to discriminate rapidly evolving traits from slowly evolving ones.

[1] K. Jacobs, *Stochastic Processes for Physicists: Understanding Noisy Systems* (Cambridge University Press, 2010).
[2] A. Cabrales, Stochastic replicator dynamics, International Economic Review **41**, 451 (2000).
